# P01-042 – Joint involvement in Armenian children with FMF

**DOI:** 10.1186/1546-0096-11-S1-A45

**Published:** 2013-11-08

**Authors:** G Amaryan, G Khloyan, R Davtyan, T Sarkissian, A Tadevosyan

**Affiliations:** 1 National Pediatric Centre for Familial Mediterranean Fever of “Arabkir” Joint Medical Centre - Institute of Child and Adolescent Health, Yerevan State Medical University, Armenia; 2 National Pediatric Centre for Familial Mediterranean Fever of “Arabkir” Joint Medical Centre - Institute of Child and Adolescent Health, Yerevan State Medical University, Armenia; 3 Centre of Medical Genetics and Primary Health Care, Yerevan, Armenia; 4 Yerevan State Medical University, Yerevan, Armenia

## Introduction

Familial Mediterranean Fever (FMF) as an ethnic disease is wide-spread in Armenia. In most cases FMF manifests in childhood. Joint involvement is the third major FMF manifestation. It usually presents as acute recurrent arthritis (ARA), arthralgia, more rare - chronic arthritis (Juvenile Idiopathic Arthritis, JIA), which are important to better learn more about the overlap between the FMF and JIA.

## Objectives

To investigate clinical and genetic characteristics of the joint manifestations in Armenian children with FMF.

## Methods

A group of 715 children with FMF was observed at the National Pediatric Centre for FMF. There were 438 boys and 277 girls, aged from 3 months to 17 years (mean age 8.64±0.17). The diagnosis of FMF was confirmed according to the generally recognized Tel-Hashomer criteria, the “Guidelines for the genetic diagnosis of hereditary recurrent fevers”(2011) and molecular-genetic detection of 12 *MEFV* mutations common for Armenians. The findings were processed with the use of standard statistical Epi-Info 2000 Program. For comparison of two nominal variables in table “two by two” Yaet’s corrected for continuity chi-square test was used, significance level p<0.05.

## Results

Joint involvement were observed in 56.4% FMF patients and manifested mainly as ARA in 30.5%, arthralgia in 21.2% and chronic arthritis in 4.7%, who also qualify for diagnosis of JIA. The risk of development of both ARA and JIA was associated with high penetrance M694V mutation, mainly M694V homozygous and M694V heterozygous genotypes. M694V heterozygous genotype was noticed significantly more frequently among FMF patients with spondylloarthropathy in compare to those without it. The probability of the development of JIA was significantly high also in heterozygotes without M694V mutation in comparison with compound-heterozygotes.

## Conclusion

The frequency of joint involvement among Armenian children with FMF (56.4%),especially in combination with JIA (4.7%), were more frequent than expected. The carriers of a single M694V mutation had more frequent arthritis, in particular JIA, spondylloarthropathy. In patients with M694V mutation chronic arthritis may be the first, early and only manifestation of FMF. We suppose, that Armenian children with all types of arthritis should be investigated for FMF. The presence of M694V mutation, in both heterozygous and homozygous genotypes, could be considered as a risk factor for arthritis, causing atypical (in heterozygotes) or severe (in homozygotes) course of FMF.

## Disclosure of interest

None declared.

